# Standardization of Epidemiological Surveillance of Group A Streptococcal Impetigo^[Author-notes ofac249-FM1]^

**DOI:** 10.1093/ofid/ofac249

**Published:** 2022-09-15

**Authors:** Kate M Miller, Jonathan R Carapetis, Thomas Cherian, Roderick Hay, Michael Marks, Janessa Pickering, Jeffrey W Cannon, Theresa Lamagni, Lucia Romani, Hannah C Moore, Chris A Van Beneden, Dylan D Barth, Asha C Bowen, for the Strep A Vaccine Global Consortium (SAVAC) Burden of Disease Working Group, Jonathan Carapetis, Chris Van Beneden, David Kaslow, Thomas Cherian, Theresa Lamagni, Mark Engel, Jeffrey Cannon, Hannah Moore, Asha Bowen, Anna Seale, Gagandeep Kang, David Watkins, Sam Kariuki

**Affiliations:** Wesfarmers Centre for Vaccines and Infectious Diseases, Telethon Kids Institute, University of Western Australia, Perth, Western Australia, Australia; Wesfarmers Centre for Vaccines and Infectious Diseases, Telethon Kids Institute, University of Western Australia, Perth, Western Australia, Australia; Perth Children’s Hospital, Perth, Western Australia, Australia; MMGH Consulting, Geneva, Switzerland; St John’s Institute of Dermatology, King’s College London, United Kingdom; Clinical Research Department, Faculty of Infectious Diseases, London School of Hygiene and Tropical Medicine, London, United Kingdom; Hospital for Tropical Diseases, University College, London, United Kingdom; Division of Infection and Immunity, University College London, London, United Kingdom; Wesfarmers Centre for Vaccines and Infectious Diseases, Telethon Kids Institute, University of Western Australia, Perth, Western Australia, Australia; Wesfarmers Centre for Vaccines and Infectious Diseases, Telethon Kids Institute, University of Western Australia, Perth, Western Australia, Australia; Department of Global Health and Population, Harvard T. H. Chan School of Public Health, Boston, Massachusetts, USA; United Kingdom Health Security Agency, London, United Kingdom; The Kirby Institute, University of New South Wales Sydney, Sydney, Australia; Murdoch Children’s Research Group, Melbourne, Australia; Wesfarmers Centre for Vaccines and Infectious Diseases, Telethon Kids Institute, University of Western Australia, Perth, Western Australia, Australia; CDC Foundation, Centers for Disease Control and Prevention, Atlanta, Georgia, USA; Wesfarmers Centre for Vaccines and Infectious Diseases, Telethon Kids Institute, University of Western Australia, Perth, Western Australia, Australia; Wesfarmers Centre for Vaccines and Infectious Diseases, Telethon Kids Institute, University of Western Australia, Perth, Western Australia, Australia; Perth Children’s Hospital, Perth, Western Australia, Australia

**Keywords:** epidemiology, impetigo, infectious disease, *Streptococcus pyogenes*, surveillance

## Abstract

Impetigo is a highly contagious bacterial infection of the superficial layer of skin. Impetigo is caused by group A *Streptococcus* (Strep A) and *Staphylococcus aureus*, alone or in combination, with the former predominating in many tropical climates. Strep A impetigo occurs mainly in early childhood, and the burden varies worldwide. It is an acute, self-limited disease, but many children experience frequent recurrences that make it a chronic illness in some endemic settings. We present a standardized surveillance protocol including case definitions for impetigo including both active (purulent, crusted) and resolving (flat, dry) phases and discuss the current tests used to detect Strep A among persons with impetigo. Case classifications that can be applied are detailed, including differentiating between incident (new) and prevalent (existing) cases of Strep A impetigo. The type of surveillance methodology depends on the burden of impetigo in the community. Active surveillance and laboratory confirmation is the preferred method for case detection, particularly in endemic settings. Participant eligibility, surveillance population and additional considerations for surveillance of impetigo, including examination of lesions, use of photographs to document lesions, and staff training requirements (including cultural awareness), are addressed. Finally, the core elements of case report forms for impetigo are presented and guidance for recording the course and severity of impetigo provided.

## DISEASE CHARACTERISTICS

Impetigo is a highly contagious bacterial infection of the superficial layer of skin. Impetigo typically begins as erythematous macules on the skin, which rapidly progress to a thin fluid-filled vesicle that becomes purulent, ruptures, and forms a thin honey-colored crust. As healing progresses, the crust thickens, tethering the underlying eroded base and becoming thinner and smaller with time. The final stages are when the crust completely resolves, and a flat, dry, hypopigmented or hyperpigmented lesion remains as evidence of recent impetigo. The healing stages take up to 30 days without treatment or up to 7 days following antibiotic treatment [1]. Exposed areas such as the face, arms, and legs are most affected, likely due to the distribution of the underlying causes of skin disruption that allow the organism to penetrate the skin. Impetigo can occur without an obvious trigger or be secondary to an insect bite, scabies infestation, minor trauma, tinea, head lice, eczema, or varicella, all of which cause a break in the skin, either directly or as a result of itching, facilitating the entry of bacteria. It is important to capture the antecedent causes of any impetigo lesion as scabies and head lice require treatment with appropriate antiparasitic agents. In contrast, tinea requires antifungals and minor trauma, and insect bites have different prevention strategies that can be activated at a public health level. See Supplementary Appendix 1 for some helpful features for identifying the likely trigger. Impetigo is caused by group A *Streptococcus* (Strep A) and *Staphylococcus aureus*, alone or in combination, with the former predominating in many tropical climates.

Strep A impetigo occurs most frequently in early childhood [2–4], with the burden of impetigo among persons varying between geographic areas. For most populations, Strep A impetigo is an acute, self-limited disease. However, in endemic settings, where Strep A is persistent in the population, it can be a current affliction with multiple lesions at various stages of resolution over months or years. Differences in the severity and frequency of impetigo between settings lead to different surveillance approaches, particularly for ascertaining cases, surveillance frequency, and epidemiological measures used to describe the disease burden.

Acute poststreptococcal glomerulonephritis (APSGN) is a potential sequela of Strep A impetigo. Contemporary studies have shown a possible association between acute rheumatic fever (ARF) and impetigo [5–7]. Recognition and treatment of Strep A impetigo is a key component of reducing the burden of these postinfectious sequelae. Effective treatment of Strep A impetigo must address the underlying scabies infection or cause of other skin lesions. Although most impetigo lesions resolve spontaneously within a month [8], systemic or topical antibiotics speed recovery, reduce transmission (to other body parts and between people), and prevent suppurative complications (eg, sepsis, skeletal infections), and may prevent nonsuppurative sequelae such as APSGN and ARF [9].

## OBJECTIVES OF SURVEILLANCE FOR STREP A IMPETIGO

An effective surveillance system for impetigo serves to (1) monitor trends in age- and sex-specific incidence or prevalence of Strep A impetigo in a population of a defined geographic area; (2) monitor trends in demographic and clinical characteristics of people with confirmed Strep A impetigo; and (3) provide estimates of disease burden of Strep A impetigo.

Potential additional objectives that may be incorporated into routine surveillance of impetigo include to (1) measure and track antibiotic susceptibility (eg, to penicillin, macrolides, clindamycin, cotrimoxazole); (2) predict or measure the effectiveness of Strep A vaccines (in development or use) or other treatment or prevention strategies; (3) monitor sequelae of Strep A impetigo (eg, sepsis, osteomyelitis, APSGN, or ARF); and (4) describe selected genotypic or phenotypic features of Strep A isolates causing impetigo (ie, *emm* types, presence of vaccine antigens, and antimicrobial susceptibility) and monitor their prevalence over time and in response to public health interventions.

Specialized (nonroutine) surveillance that has the capacity to conduct concurrent throat culture in addition to serial swabbing may seek to understand transmission dynamics and the interaction with Strep A throat carriage and whether this is important in ongoing cycles of skin infection.

## CASE DEFINITIONS AND FINAL CLASSIFICATION

Case definitions are important for standardizing data collection and are the foundation of disease surveillance, enabling comparisons of surveillance data across jurisdictions and monitoring the impact of interventions. Impetigo is a clinical diagnosis made by surveillance team members with the necessary education, training, and experience to recognize skin sores. The definitions and classifications in Table 1 can also be used as clinical endpoints for vaccine efficacy trials and postlicensure effectiveness studies.

**Table 1.** Case Definitions and Classifications of Impetigo for Surveillance

**Figure 1.**
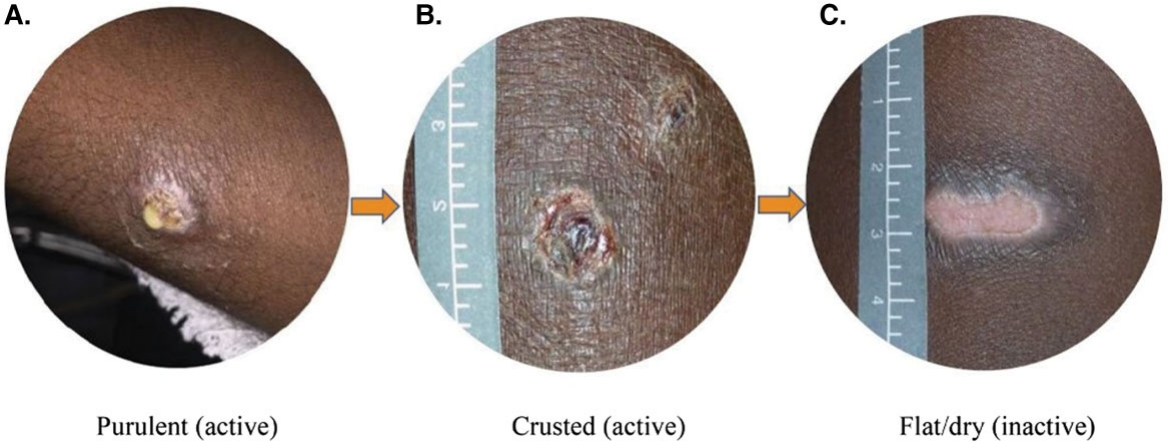
Classifications of impetigo: purulent (*A*); crusted (*B*); flat/dry (*C*).

**Case Definitions^a^**
***Clinical bullous impetigo:*** Presence of ≥1 skin sore, defined as a large fluid-filled blister of 1–2 cm, usually in areas with skin folds such as the armpit, groin, between the fingers or toes, beneath the breast, and between the buttocks.
***Clinical nonbullous impetigo:*** Presence of ≥1 skin sore, defined as a round papular, pustular, or ulcerative lesion of 1–2 cm.
***Strep A (Streptococcus pyogenes) impetigo:*** Defined as clinical nonbullous impetigo (as defined above) with the isolation of Strep A in culture from an active impetigo lesion [10].
**Case Classifications**
***Incident case of Strep A impetigo:*** Defined as an active lesion from which Strep A is grown in culture but which occurs in a person who did not have any active lesions in the past 14 days (over a long surveillance period, this could occur more than once for each person) [10].
***Prevalent case of Strep A impetigo:*** Defined as 1 or more pustular or crusted lesions from which Strep A is grown in culture.
***Active (ongoing):*** Defined as presence of round or linear papular, pustular, ulcerative, or crusted lesions of 1–2 cm, which may be surrounded by erythema and/or the presence of frank pus (Figure 1*A*and 1*B*). In this situation, additional culture confirmation is necessary.
***Inactive (resolved):*** No active lesions but evidence of flat, dry lesions on the surface of the skin that are healing (Figure 1*C*). In areas where impetigo is endemic, a person may have new lesions frequently appearing while others resolve.

a Clinical bullous impetigo is a bacterial skin infection caused only by *Staphylococcus aureus*, whereas nonbullous impetigo is caused by Strep A or *S aureus* and is thus the focus of this surveillance protocol.

## MICROBIOLOGICAL TESTS USED TO DETECT STREP A AMONG PERSONS WITH IMPETIGO

Diagnosis of Strep A impetigo is confirmed by culture of the bacteria from a specimen obtained from an active lesion. Routine microbiological culture of skin specimens is done in a laboratory setting to inform diagnosis and for antimicrobial susceptibility testing. Typically, clinical skin swabs are inoculated onto blood agar plates; however, selective plates can be used [11]. Inoculated agar plates are initially incubated at 37 °C for 18–24 hours, but incubation up to 48 hours may be necessary. The addition of 5%–10% carbon dioxide for incubation may enhance growth but is not essential. Following incubation, plates are inspected for β-hemolytic colonies to undergo subculture purification and confirmation with further biochemical tests including latex agglutination testing (for Lancefield groups A, C, G), bacitracin sensitivity, and pyrrolidonyl arylamidase testing. No biochemical test is 100% specific for *S pyogenes* [12] and so tests are frequently used in combination. Purified colonies can be stored enabling further testing, with long-term storage between −70°C and −80 °C in a suitable cryoprotectant medium (eg, in Todd-Hewitt glycerol broth or skim milk tryptone glucose glycerol broth [STGGB]). Molecular profiling of *emm* types (via Centers for Disease Control and Prevention methodology or whole genome sequencing [WGS]–derived methods [13]), or WGS to differentiate Strep A strains, can support surveillance by indicating the diversity of strains in a population over time and to map transmission in communities.

All surveillance should seek to identify clinical cases of impetigo based on the presence of any purulent or crusted lesion, encouraging culture of the most purulent impetiginous lesion per person with active impetigo. During early infection, culture of the exudate or pus from an impetigo lesion can identify the presence of Strep A. Shortly after the pus presents, it thickens and hardens to form a crust, and a culture can be obtained by gently rolling a moistened swab over the crust. The swabbing technique used to obtain culture should be standardized.

In the future, microbiological diagnosis using molecular methods such as polymerase chain reaction may be used.

### Specimen Collection

#### Equipment and Supplies

The following equipment and supplies are needed. (1) Gloves (need not be sterile); (2) Sterile swabs (cotton wool or synthetic fiber) and culture medium (eg, eSwab, STGGB, Amies); (3) Sterile normal saline; (4) Biohazard plastic bags, or clean plastic bags that can be labeled; (5) Transport container; and (6) Cooling bricks (if refrigerated storage is recommend for choice of culture medium).

#### Methods of Sample Collection

1. Verify the identity of the person and label a sterile culture swab tube with the information requested by the protocol (typically 2 case identifiers, such as participant initials and surveillance number, the date, and an identifier for the person plating the organism) and complete a sample form with participant details.
2. Perform hand hygiene, and if appropriate put on gloves.
3. If a person has multiple lesions, it is recommended that the surveillance staff swab the most purulent lesion; older lesions are more likely to be coinfected with *S aureus* or fail to grow any organisms.
4. Use a sterile swab (packages with intact wrapping), taking care to keep the swab tip sterile after the packaging has been opened.
5. Choose the most purulent lesion to swab if a person has multiple lesions.
6. The sample collection method depends on the character of the impetiginous lesion:
  - For purulent lesions, pass the swab over the wound, collecting available pus.
  - For crusted lesions without evidence of pus, the swab should be moistened with sterile water or saline and rolled gently backwards and forwards several times over the crust.
  - For dry nonvesicular lesions without a crust, the swab should be moistened with sterile water or saline and rolled backwards and forwards gently over the base of the lesion several times.
7. Avoid touching normal skin with the swab.
8. Put the swab into the swab tube and make sure the top is screwed on or pushed on firmly in place.

#### Storage and Handling

The following storage and handling should be taken. (1) All specimens should be stored in sealed biohazard plastic bags or inside a biohazard-labeled sealed container: store at the temperature required by culture medium. For example, room temperature storage is suitable for eSwabs (Copan, Italy), whereas refrigerated (in fridge) conditions are recommended for specimens stored in STGGB. (2) Sample collection documentation must be kept with specimens, but not in the same compartment in case of leakage.

#### Documentation

The following documentation procedures should be taken. (1) Label all specimens: follow instructions on sticky label on tube/swab container; and minimum information needed: unique participant ID number, date specimen collected, and exactly what specimen is (eg, blood, wound swab). (2) A specimen transport log form should be used, consisting of: place, date, and time of collection shipment; and contents of shipment including participant ID numbers, specimen types, and order of storage.

#### Specimen Transfer

The following procedures should be taken for specimen transfer. (1) Place absorbent material in sealed biohazard bags with specimens in case of sample leakage. (2) Put into recommended portable transport container. For samples collected into storage medium with refrigeration recommended (ie, STGGB), store sealed bags in between ice cooler bricks. (3) Seal lid of portable container as instructed or with waterproof tape. (4) Label all containers clearly with: place, date, time of packing, and destination; and biohazard sticker (if no sticker, write it in big letters using black marker). (5) Make sure the courier knows what contents are, so they will not be left in a hot place and will be promptly delivered to the laboratory. (6) Specimens should reach the laboratory as soon as possible (≤10 days).

## TYPES OF SURVEILLANCE

The selection of surveillance strategies depends on the burden of skin sores within the community (endemic vs nonendemic settings), surveillance objectives, the surveillance location, services accessibility, and the resources available to conduct surveillance (see Supplementary Appendix 2 for key surveillance definitions). In endemic settings with a high burden of impetigo, skin infections are prevalent and recurrent. It is common for parents, caregivers, and healthcare providers to regard skin infections as a self-limiting and minor illness in these settings. As a result, many people may not access healthcare services for impetigo, with skin infections subsequently left untreated or only treated by healthcare providers in conjunction with other health presentations. In situations where impetigo is normalized [14], enhanced surveillance is recommended. Minimal surveillance is appropriate when a minimum estimate of disease burden is considered adequate for surveillance purposes, the population at risk is well-characterized demographically, and bias toward more severe cases is acceptable for the surveillance being undertaken [15]. Minimal and enhanced surveillance strategies for Strep A impetigo are described in Table 2 to provide guidance for those with limited resources and those with greater capacity, respectively.

**Table 2.** Surveillance Strategies for Group A Streptococcal Impetigo

*Minimal Surveillance*
Limited to passive surveillance of primary healthcare facilities Based on clinical signs and symptoms or a diagnosis recorded in health facility databases and microbiological data from laboratory databases. Settings include primary healthcare clinics (eg, outpatient clinics and doctor’s offices), hospitals, and clinical laboratories. In endemic settings, mobile health services at defined posts, additional fixed health posts, and community-based programs where people seek care at their discretion can also be utilized. Participants are those who present to healthcare or other relevant settings on their own accord. If the provider or surveillance officer determines that the case definition for impetigo has been met, it can be recorded in the EMRs or in a report provided to the surveillance system or local public health authorities. Standard case report forms may be provided to the health facilities or laboratories for completion and submission to the surveillance program.
*Enhanced Surveillance*
Includes active case finding and laboratory confirmation among a defined cohort with regular follow-up for a defined period Active surveillance is the preferred method for optimizing case detection of impetigo. Active surveillance can also be helpful in LMICs where the population cannot easily access health services, limited staff is available, and diagnostic testing is not universally available. Settings include households, early childhood centers/schools and primary healthcare clinics. Well-defined clinical practices and laboratory methods are established prior to surveillance and remain constant throughout the surveillance period. Active surveillance requires timely detection of new cases to ensure appropriate testing is conducted to confirm Strep A culture from an active lesion. Participants are followed prospectively, ideally weekly or fortnightly, for a defined period across seasons using standard methods to collect demographic and clinical information and microbiological testing to confirm Strep A cases. Audits are performed biannually to assess the completeness of case ascertainment, accuracy, timeliness, and laboratory performance. Regular feedback of data/information is provided to healthcare workers and others involved in the surveillance process. This critical communication engages healthcare workers in the process and informs their clinical practice.

Abbreviations: EMR, electronic medical record; LMICs, low- and middle-income countries; Strep A, *Streptococcus pyogenes*.

A quality management plan should be written before the start of surveillance to establish and ensure the quality of processes, data, and documentation associated with surveillance activities. Moreover, all surveillance should be conducted in accordance with ethical guidelines (Supplementary Appendix 3).

## CASE ASCERTAINMENT AND SURVEILLANCE SETTINGS

For each data source, surveillance staff should (1) know the purpose of the data source and whether data have been routinely collected as part of patient care, mandatory collection of data under legal mandates, collected for research purposes, or other; (2) identify any legal mandates governing the operations of the data source that may affect the accessibility or quality of data from that source; and (3) describe the representative population for the data. Case ascertainment may be active or passive (Supplementary Appendix 4).

### Households

Household screening is effective for surveying large numbers of community members across a wide age range [16]. Household screening can identify skin sores in persons who are unable to attend school, do not seek healthcare, or are unable to due to lack of time, financial constraints, or accessibility issues. Population-based household surveillance reduces the bias arising from inequalities in access to school and healthcare but is time intensive, resource intensive, and costly. Local and cultural schedules and customs will need to be considered to maximize the impact of the household surveys.

### Early childhood centers/schools

Schools provide a practical setting for disease surveillance, given that most surveillance programs are expected to include school-aged children, and offer the logistical advantage of many children in a single location. Where feasible, surveillance should be complemented with settings that capture younger children, such as early childhood centers and mother and child clinics, recognizing that babies often have the highest burden [17].

Certain biases and sampling frameworks need to be considered within school settings to estimate the disease burden accurately. For example, surveying school attendees will lead to selection bias in a population with high levels of school absenteeism, resulting in an underestimate of disease burden. Factors associated with school nonattendance may include impetigo itself and conditions associated with the risk of impetigo such as poverty and/or ill health. The bias should be acknowledged and school attendance rates cited. If possible, school nonattenders should be surveyed, although this is more difficult and costly. Another potential bias may result from a clustering effect. For example, the risk of impetigo in children attending a particular school may differ from those attending another school due to differences in geographical location and/or other demographic and social factors. Similarly, transmission can occur within a classroom, so sampling 1 school or classroom will not give a representative or generalizable estimate. Thus, surveillance should ideally cover all schools within a defined population and multiple classrooms within a school; this will increase costs but also increase external validity. Options include surveying all children or surveying a subset of children in each school, potentially increasing the number of schools included. For the latter, an appropriate method of selection within schools should be chosen, for example a random sample of children across classes to remove the possibility of a clustering effect by class.

### Primary healthcare

Primary healthcare surveillance can be centrally located and cover many people, possibly an entire community/population. Active surveillance using primary healthcare clinics may comprise of recruiting participants registered at the clinic, requesting that they present to the clinic upon developing predefined impetigo symptoms. The symptoms should be sufficient to warrant a visit to the primary healthcare clinic (ie, discrete sores/lesions with pus or crusts). The surveillance staff would regularly reach out to families, ideally weekly, during the surveillance period to facilitate presentation to primary healthcare clinics and record new lesions. Primary healthcare surveillance relies on engagement from surveillance staff and primary practitioners and healthcare workers to maintain adequate retention rates, particularly for prospective longitudinal surveillance [18, 19].

Passive surveillance of impetigo cases among persons in primary healthcare settings involves recording data on patients who present to primary healthcare clinics; however, these patients may not represent all people with impetigo in the community. For example, due to the often-mild symptoms of impetigo and normalization of the disease in some settings [14], not all people with impetigo attend primary healthcare facilities, and the demographic and clinical characteristics of those who do may not be representative of all cases or the underlying burden in the community [20]. Primary healthcare surveillance can also be limited by physician recognition and documentation of skin infections, which may be low in high-burden settings [14].

Surveillance can be optimized by predefining a data collection protocol before starting the surveillance period that specifies case definitions, data requirements, and the criteria/process for obtaining cultures. As access to laboratory facilities and the propensity to seek laboratory confirmation will naturally vary between physicians, a predefined protocol will improve data completeness and uniformity for obtaining bacterial cultures from skin sores to confirm Strep A.

## SURVEILLANCE POPULATION

A surveillance protocol should clearly describe enrollment eligibility criteria. Most protocols would benefit from surveying children aged 0–11 years; however, age eligibility can vary between sites, depending on local needs and capacity. Persons receiving prophylactic antibiotics for any cause and those with underlying immunocompromising conditions or chronic diseases should not be excluded from surveillance.

The surveillance population is the total number of eligible at-risk people from which cases of impetigo are identified. This population, or denominator, must be well-characterized to derive meaningful disease burden estimates. Without an accurate account of all people in the population who could potentially be evaluated for Strep A impetigo, disease estimates may be under- or overestimated [21, 22].

Some settings allow population-wide data on disease burden to be recorded and analyzed. Examples include household surveillance in a representative sample in a community or a healthcare setting that serves an entire community. In these cases, the surveillance population would be defined as all eligible people who reside in the community. If government-derived census data are used to derive the community’s demographic profile, such as the number of people in relevant age categories, the accuracy of the data must be assured.

In instances where select primary healthcare facilities serve a portion of a population residing in the geographical catchment area, healthcare utilization surveys can be used to estimate the denominator corresponding to the cases of interest, improving the accuracy of disease burden estimates and enabling rate calculations [23]. The denominator is the number of patients within the geographical catchment area who would be expected to attend that primary healthcare facility if signs and symptoms of Strep A impetigo develop. Cases not residing in the defined catchment area should be excluded. Ideally, the denominator population should be defined before surveillance begins.

When undertaking surveillance in a sample of schools and/or classrooms, the surveillance population is the number of children who agree and have parental or guardian appropriate consent to participate in surveillance. The results can be generalized to the entire community if schools and classes are randomized at the start of surveillance or appropriate demographic characteristics of participants can be weighed against the characteristics of the catchment population.

## SPECIAL CONSIDERATIONS FOR IMPETIGO SURVEILLANCE

### Administrative database review

Codes used to identify impetigo in electronic medical records are shown in Table 3. Note that the codes are not pathogen-specific and therefore represent a clinical diagnosis of impetigo rather than a microbiologically confirmed diagnosis of Strep A impetigo. See Supplementary Appendix 5 for considerations for using administrative health databases in surveillance.

**Table 3.** Specific Codes for Impetigo in Electronic Medical Record Databases

Type of Healthcare System	Impetigo Code
Primary healthcare system	
*International Classification of Primary Care, version 2* (*ICPC-2*) system	S84
Read system	M05
SNOMED CT	48277006
Hospital data system	
*International Statistical Classification of Diseases and Related Health Problems, Tenth Revision* (*ICD-10*) [24]	L01.0, L01.1, L08.0 (if grouped with pyoderma)

Abbreviations: CT, clinical terms; SNOMED, systematized nomenclature of medicine.

### Examination of lesions

For clinical examination of patients presenting for diagnosis and treatment of skin infections, the entire skin surface may need examination. The recommendation is first to examine the hands and arms, feet and legs, neck, face, and scalp (ie, visible body surfaces). Then ask the person whether there are any skin lesions on the trunk or buttocks and examine these areas if appropriate. Conditions for an appropriate examination include a private room or privacy screen, with a chaperone present.

For surveillance assessments, we recommend examining visible body surfaces only, as most impetigo lesions occur on these areas. If personally and culturally appropriate and privacy allows—as stipulated in the surveillance standard operating procedures—and the participant indicates the presence of skin sore(s) on the trunk or buttocks, these areas should be examined and documented.

Scabies infestations often co-occur with impetigo. Scabies lesions can be identified clinically as the presence of a burrow or papule that is itchy, in the context of other contacts having similar lesions [25]. Diagnostic certainty of scabies in fieldwork is not currently possible in most low- and middle-income country settings [26]. In 2020, the International Alliance for the Control of Scabies released Consensus Criteria for the Diagnosis of Scabies that could be applied to various clinical, research, and public health settings and facilitate surveillance [25]. The itch of scabies may introduce secondary bacterial infection with the appearance of purulent or crusted scabies.

### Treatment

Active lesions must be treated as part of the surveillance protocol and consistent with local treatment guidelines or developed in consultation with local health providers. Where identified, scabies lesions also need treatment as per local protocols.

### Training and supervision

Prior training and supervision are needed for accurate data collection, which depends on the clinical acumen and experience of the surveillance staff and/or community healthcare workers conducting the assessments. Skin infection recognition is important to identify skin infections correctly. Underrecognition or normalization of skin infections can be an issue in endemic settings. Targeted upskilling or refresher training should occur before each surveillance visit to improve the identification, assessment, and classification of skin conditions.

Formal off-the-job training can be provided via face-to-face workshops, online training manuals and videos, and/or self-assessment materials using a written guide and photographs. Local community healthcare workers in field settings can also be paired with experienced clinicians and/or appropriately trained surveillance staff to supervise clinical assessment and diagnosis of eligible people. On-the-job training can occur for a defined period or until a desired level of competency is achieved.

### Resources

The Integrated Management of Childhood Illness in-service training guide is a useful resource for developing training [27]. Training materials should be readily available online for easy access and in paper format for field settings where internet connectivity is unreliable. Resources could include a participant manual comprising background information on skin condition diagnosis, photograph booklet, and example group exercises and role plays. Training modules should be updated regularly.

“Recognizing and Treating Skin Infections: A Visual Clinical Handbook” [28] can be used to train healthcare professionals in diagnosing impetigo. An online quiz is also available for training purposes (https://infectiousdiseases.telethonkids.org.au/resources/skin-guidelines/).

Several interactive mobile applications have been developed to facilitate the timely diagnosis and treatment of skin diseases, including impetigo, such as the World Health Organization skin neglected tropical diseases application (Skin NTDs App), VisualDx, and the No Leprosy Remains application (SkinApp NLR).

### Photographs

Capturing digital images of impetigo can play an important role in surveillance. When participants provide consent, photographs can be used to document lesions, measure treatment outcomes (monitor sore healing in response to antibiotic treatment), and provide clinical support (for experienced clinicians to provide diagnostic support to surveillance staff in the field), and for training purposes (assist in recognizing skin infections and improving diagnostic classification by surveillance staff) and quality control (validate diagnostic classification; eg, a random sample of digital images of skin infections together with the diagnosis made by surveillance staff can be reviewed retrospectively by experienced clinicians).

A standardized protocol for capturing digital images of impetigo [29] should be followed to ensure that the entire sore is captured in sufficient detail, to determine the margins and most of the interior and ensure the uniform collection of digital images. A unique identifier should be used to label photographs, rather than personal identifying information, to ensure visual data safety (Supplementary Appendix 6).

### Cultural awareness training

Cultural awareness training should be included in the education and training program to ensure respectful practices are maintained and that cultural protocols are followed.

### Community engagement

Community engagement helps provide a considered approach to surveillance (Supplementary Appendix 7) and ensures that the project has community value. It also ensures that community members have an opportunity to clearly express their values and concerns and develop a degree of ownership. The time required to forge relationships between surveillance staff and communities should not be underestimated and must be built into the surveillance protocol at the outset.

The level of community engagement in the design, implementation, monitoring, and evaluation of surveillance will depend on available resources and community capacity. Key stakeholders include community leaders, teachers, health staff, and volunteers.

### Frequency

The schedule of passive surveillance will be dictated by the pattern of case presentation at the surveillance site(s). In endemic settings, the number of prevalent cases is expected to be relatively constant in the absence of an intervention program; however, conducting surveillance visits across multiple seasons is recommended. To accurately identify incident impetigo cases (see “Case Classifications” above), people under active surveillance in nonendemic settings will ideally be visited at least once a month to detect new cases, determine the duration of lesions, and document any clearance of lesions.

### Period of surveillance

Defining the surveillance duration depends on the availability of resources to support the surveillance system and the time needed to achieve the surveillance objectives. A minimum of 1 year is recommended due to the influence of seasonality and the potential for intermittent impetigo outbreaks (short-term surveillance may not accurately depict long-term burden). Several years of surveillance will elucidate year-to-year variations in incidence and M or *emm* type distribution and help monitor the impact of public health interventions.

### Season

Seasonal variation in the incidence of Strep A impetigo occurs in temperate areas worldwide, with peaks in the rainy, hot months. If possible, surveillance staff should perform surveillance across all seasons to capture the changes in disease prevalence over time. In areas where seasonality is well-described, limiting surveillance to months when most cases are likely to occur has efficiencies but will produce inflated prevalence estimates and annual incidence rates. Continuous surveillance over 12 months is optimal but may not be possible due to school holidays, extended absences from school to tend to farms or other family or community duties, lack of access to remote areas during wet seasons, and closure of communities for cultural reasons.

### Measurement of disease burden

The burden of Strep A impetigo can be described in terms of incidence or prevalence. For nonendemic settings, the incidence of impetigo (number of new cases occurring in a specified period) is the preferred epidemiological measure to describe burden due to the relatively short duration of disease. For endemic settings, the prevalence of impetigo (number of cases that exist at a given point in time) may be more appropriate and practical due to the frequent recurrence of skin infections and large number of lesions on the body surface. However, tracking incidence over time should be considered if cases are expected to be appropriately treated or other intervention strategies are implemented (such as vaccine trials) (further detail on disease burden measurements are provided in Supplementary Appendix 8).

## DATA COLLECTION AND CASE REPORT FORMS

### Consent

Before initiating an assessment and collecting data or specimens, consent for participation in the surveillance program may need to be obtained based on the determination of an institutional review board. For children, consent needs to be obtained from the parent or legal guardian, and before examining, permission (assent) must be obtained from the child. Consent should be voluntary and based on sufficient information and an adequate understanding of the surveillance program and the implications of participation. Flipcharts and interpreters may help improve information delivery so that participants are clear about what they are consenting to. If consent is not obtained, do not proceed. For prospective active surveillance programs, each participant must be informed that participation in the project is voluntary and that they are free to withdraw, without justification, from the surveillance system at any time without consequences. Note that the age at which consent can and should be given by the child will vary between countries/jurisdictions. It is the responsibility of surveillance staff to confirm the requirements of local, regional, or national authorities. Informed consent may be obtained for surveillance, skin photographs, skin swabs, and storage of swabs for future use including genetic sequencing and transcriptome analysis.

### Case Report Forms

Case report forms should be based on collecting only the information required to achieve the surveillance objectives. See Supplementary Appendix 9 for a list of recommended and optional variables for inclusion in all case report forms. Case report forms can be paper based but, increasingly, secure electronic data forms are used. Electronic case report forms offer several benefits such as early detection of cases and timely information flow, relatively inexpensive operating costs, and improved data quality (accuracy and data completeness) via imbedded validation checks.

General surveillance information includes unique identifier, date and time of first enrollment or specimen collection, and site where participant is seen, such as setting, location, postcode, state/province/region, and country. Each encounter should also record a surveillance visit number/episode number if repeated episodes from the same person are included.

Key demographic information includes date of birth or, if date of birth not available, age in days or months if <12 months and otherwise in years, sex, ethnic origin/race, residential postcode, state, and country.

Clinical and epidemiologic information includes lesion type and number of lesions, likely trigger, signs and symptoms, duration and pain related to lesions, epidemiologic risk factors, treatment details, and microbiological variables.

### Course Of An Episode

Depending on the objectives of the surveillance program, it may be useful to count and document the total number of discrete sores for each person. Sores can be further classified as flat/dry (resolved, healed, or inactive impetigo), crusted (scab over a skin sore, active impetigo), or purulent (wet or moist with obvious presence of pus, active impetigo) (Figure 1). A body map for the recording of lesions is shown in Supplementary Appendix 10. For surveillance programs concerning the course of the episode following diagnosis or treatment, the outcome measures may relate to the duration of an episode or sore healing. Only those who suffer an episode of impetigo contribute information to this analysis. For example, the mean duration per episode of impetigo could be calculated for each person.

#### Sore Score

The severity of impetigo affecting those with residual lesions can also be recorded. A severity score considers types of sores, number of regions affected on the body, number of lesions, and the presence of any complications (ie, boils/abscess, cellulitis). Table 4 provides an example severity grading scale for impetigo, with a numeric score for each person. The score is calculated by adding values for sore types present anywhere on the body (3 = purulent; 2 = crusted; 1 = flat/dry; maximum of 6 points per person) to those for the number of body regions affected (1 = 1 region; 2 = 2 regions; maximum of 3 points per person for 3 regions), sore numbers (3 = >20 lesions; 2 = 5–20 lesions; 1 = 1–4 lesions; maximum of 9 points per person), and the presence of any complications (3, for complications). Note that the cutoff values used to define categories for sore numbers may differ between settings; they should be validated for the population under surveillance before recommending their use as a primary endpoint in an intervention trial [30].

**Table 4.** Impetigo Severity Sore Score

Category	Score			Maximum Score per Person per Category
	1	2	3	
Type of sores	Flat/dry	Crusted	Purulent	6
Body regions affected	1	2	3	3
No. of lesions				
Arms (upper limbs)	1–4	5–20	>20	3
Legs (lower limbs)	1–4	5–20	>20	3
Neck/scalp/face	1–4	5–20	>20	3
Presence of complications	…	…	Yes	3
Maximum total score per person				21

Adapted from Carapetis et al [30].

## Supplementary Data

Supplementary materials are available at Open Forum Infectious Diseases online. Consisting of data provided by the authors to benefit the reader, the posted materials are not copyedited and are the sole responsibility of the authors, so questions or comments should be addressed to the corresponding author.

## Supplementary Material

ofac249_Supplementary_Data
