# Supplementary material for: Standardization of Epidemiological Surveillance of Group A Streptococcal Impetigo
Source: Open Forum Infect Dis. 2022 Sep 15;9(Suppl 1):S15–24. doi: 10.1093/ofid/ofac249 (PMC9474945; doi:10.1093/ofid/ofac249)
Supplement: ofac249_Supplementary_Data [file ofac249_supplementary_data.docx]

**Standardization of Epidemiological Surveillance of Group A Streptococcal Impetigo**

Supplementary Appendices

Table of Contents

[Appendix 1: Common Triggers of Impetigo 2](#_Toc112316230)

[Appendix 2: Definition of Key Surveillance Terms 3](#_Toc112316231)

[Appendix 3: Good Practice and Ethical Considerations 4](#_Toc112316232)

[Appendix 4: Comparisons of Advantages and Disadvantages of Active and Passive Surveillance 5](#_Toc112316233)

[Appendix 5: Administrative Health Databases 6](#_Toc112316234)

[Appendix 6: Quick List for Capturing Standardized Photographs 7](#_Toc112316235)

[Appendix 7: Benefits from Working with Communities to Conduct Surveillance 8](#_Toc112316236)

[Appendix 8: Terms to Describe Disease Burden 9](#_Toc112316237)

[Appendix 9: Variables for Inclusion in Impetigo Surveillance Datasets 10](#_Toc112316238)

[Appendix 10: Body Map to Record Skin Sores from Surveillance Assessments 12](#_Toc112316239)

[References 13](#_Toc112316240)

## Appendix 1: Common Triggers of Impetigo

| **Trigger** | **Key Features** |
| --- | --- |
| Non-infected scabies | Itchiness AND papules often between fingers and toes, on wrists, elbows, knees, ankles, and bottom. Babies often have ‘pimple-like’ pustules on the head, hands, and feet. |
| Infected scabies | Itchiness AND papules AND lesions with pus or crusts, usually on hands, elbow, armpits, under breasts, buttocks, and feet. |
| Tinea | Scaly, itchy well-defined patches on skin. Skin may appear lighter or darker and tougher than usual. Any part of the body can be affected. Tinea may also affect the nails (thickened, deformed, crumbly nails) or scalp (thickened round lesion with broken hair). |
| Head lice | Itchiness of the scalp. Moving adult lice can often be seen using a good light. Brown or white eggs may be stuck on hairs, particularly near the scalp. Purulent and crusted lesions of the scalp may develop due to scratching. |
| Eczema | Itchiness AND inflamed patches of skin. Chronic eczema can cause the skin to appear thick, scaly, flaky, and dry. |
| Minor trauma | May include cuts, abrasions, and minor superficial wounds. Minor trauma becomes secondarily infected with features of pain, surrounding erythema, purulence, and thickening crust. |

## Appendix 2: Definition of Key Surveillance Terms

| **Syndromic surveillance** | Syndromic surveillance refers to the use of a clinical syndrome (a constellation of symptoms and signs) as the case definition for detection of suspect cases. Syndromic surveillance can be used for initial case detection, but laboratory confirmation should occur to increase the accuracy of the system.^1^ |
| --- | --- |
| **Active surveillance** | Active case detection means that designated public health surveillance staff are directly involved in detecting cases.^1^ |
| **Passive surveillance** | Passive case detection means that health facility staff detect and report cases to the public health system.^1^ |
| **Facility-based surveillance** | Facility-based surveillance is based on ascertainment of cases in persons who seek care at health facilities, including outpatient clinics, doctors’ offices, hospitals and emergency departments.^1^ |
| **Sentinel-site surveillance** | Sentinel-site surveillance refers to a system that captures cases at one or more specialized sites, such as hospitals, clinics, schools or pharmacies.^1^ |
| **Community-based surveillance** | Community-based surveillance is the systematic detection and reporting of events of public health significance within a community-by-community members. Community-based surveillance enables earlier detection of the disease of interest and captures illnesses in persons who do not seek care in a hospital.^2^ |
| **Population-based surveillance** | Population-based surveillance attempts to capture all cases in a well-defined catchment population (for example, the entire population of a country). |
| **Healthcare utilization surveys** | Healthcare utilization surveys seek to characterize the health care-seeking behavior of ill persons by describing where ill persons sought health care for their illnesses, and soliciting reasons for not seeking health care.^3^ |
| **Unique identifier** | Unique identifiers are unique numbers or numbers and letter combinations that are allocated to a specific individual person. |

## Appendix 3: Good Practice and Ethical Considerations

**Monitoring/Audit**

A systematic and independent audit of surveillance systems should be undertaken to ensure that surveillance and surveillance-related activities were conducted following the relevant surveillance protocol, SOPs, ethical guidelines, and regulatory requirement(s) established by local public health. Existing surveillance review tools can be modified to guide the investigation (e.g., WHO’s ‘[Tools for a surveillance review: Vaccine Preventable Diseases Surveillance Standards](https://www.who.int/publications/m/item/vaccine-preventable-diseases-surveillance-standards-annex1)’). Surveillance as part of a clinical study should adhere to the International Council for Harmonisation of Technical Requirements for Pharmaceuticals for Human Use (ICH) Guidelines for Good Clinical Practice.^4^

**Quality control and quality assurance**

A quality management plan should be written before the start of surveillance to establish and ensure the quality of processes, data, and documentation associated with surveillance activities. It encompasses both quality control (QC) and quality assurance (QA) activities.

Surveillance systems should develop SOPs to ensure confidentiality for all cases, ensure that clinical specimens and bacterial isolates obtained are not compromised by human and processing errors, validate data integrity, and maintain multiple layers of security. A SOP will ideally detail:

- Data storage. Including participants’ unique surveillance ID numbers in each respective dataset enables linkage to other datasets, such as hospital admissions, facilitating the capture of complications and ensuring that all personal identifying information is removed from research/surveillance datasets.
- Data evaluation for protocol compliance and source document accuracy.
- Document review (e.g., specimen tracking logs, questionnaires), who is responsible, and frequency.
- Who the responsible person is for addressing QA issues (correcting procedures that do not comply with the surveillance protocol) and QC issues (correcting errors in data entry).
- Staff training activities and processes for documenting surveillance staff training.
- Maintenance and strict adherence to surveillance delegation log (list of staff involved in the surveillance and their duties/roles).
- Clinical and laboratory SOPs and accreditation.
- Regular audits of surveillance data to ensure accuracy and completion.
- System for periodic and refresher training for surveillance team.

**Ethics of surveillance**

The global network of WHO Collaborating Centers for Bioethics in collaboration with the U.S. Centers for Disease Control and Prevention developed ethical guidelines for public health surveillance, including common good, respect for persons, and good governance. The guidelines cover the (i) broad responsibility for undertaking surveillance and subjecting it to ethical scrutiny; (ii) obligation for ensuring appropriate protection and rights; (iii) considerations in making decisions about how to communicate and share surveillance data. The guidelines are available at <https://apps.who.int>. Countries should implement these guidelines and monitor them regularly. As appropriate, surveillance protocols should adhere to existing country-specific ethical guidelines.

## Appendix 4: Comparisons of Advantages and Disadvantages of Active and Passive Surveillance

| **Advantages** | **Disadvantages** |
| --- | --- |
| ***Active surveillance*** |  |
| - Sensitive system that facilitates early detection of new cases. Early detection allows surveillance to contribute to the prevention of post infectious sequalae - Higher case ascertainment rate - More accurate identification of cases - Ability to verify information in the case of missing data or suspected data entry errors - Data collected can be comprehensive and specific to the surveillance objectives - Can evaluate the quality and effectiveness of case-finding process, minimizing selection bias - Allows real time analysis and ability to respond/modify approach to surveillance and care - Can promote disease awareness and good health practices | - Can be costly and resource intensive - Requires dedicated surveillance staff and/or extensive training and upskilling - Can be demanding on surveillance sites - Barriers to accessing communities (i.e., distance/cultural barriers) |
| ***Passive surveillance*** |  |
| - Can be conducted retrospectively - Requires fewer resources than active surveillance - Inexpensive and can cover large areas | - Responsibility for reporting new cases lies with healthcare workers/laboratory staff; thus, it can be difficult to ensure consistency of reporting by healthcare providers and other reporters - Difficulties caused by lack of standardization in terms of case definitions and coding - Tends to under-report disease - Commonly associated with incompleteness of data recording or microbiological studies - Often difficult to confirm data recording or entry errors retrospectively |

## Appendix 5: Administrative Health Databases

Administrative data from laboratory datasets and electronic medical records (EMRs) from primary healthcare and emergency departments covering whole communities can provide a timely and cost-effective surveillance option.

An important consideration when using EMRs to calculate disease estimates in a population is that the data are collected and coded as part of service delivery rather than for surveillance purposes. As such, EMRs are often prone to missing data on key fields and require the conversion of unstructured/narrative text, which can be resource-intensive and subjective. For EMRs that include or rely on free text, new methods in machine learning or deep learning could improve case identification.^5,6^ Data from EMRs are limited to patients who attend health services and are subject to variance in physician’s propensity to seek microbiological confirmation, which may be subject to bias (e.g., more severe infections, more clinically ambiguous, one not responding to treatment) and underestimate disease incidence. However, an advantage to administrative data is that, in well-established systems, data are collected systematically, well-structured and are often population-based. EMRs can form the basis of enhanced surveillance by using an additional data collection form to augment routinely collected data.

Routinely collected clinic data may be insufficient for evaluating potential cases against the full criteria required to meet surveillance case definitions, especially when microbiological testing is not routinely conducted or recorded. Further, the data may be insufficient for addressing other surveillance objectives, such as variant typing and antimicrobial susceptibility testing.

EMR databases can vary in the number of diagnoses that can be recorded. Many hospital admission databases contain a principal or primary diagnosis as the main reason for hospital admission and several additional diagnosis codes. Impetigo may be recorded as an additional diagnosis rather than the principal diagnosis, as was the case in a population-based data linkage study in Western Australia where 41% of all skin infection hospital admissions only had skin infection ICD-10 diagnosis codes in the additional diagnosis fields ^7,8^. Consequently, analysts should check all diagnosis fields, with secondary diagnosis or comorbid cases of impetigo (i.e., not principal diagnosis) considered a case and included in the numerator.

A lack of clinical documentation on skin infections in the hospital records or misdiagnosis in outpatient facilities where impetigo is less common may contribute to underestimating the true burden of skin infections.^9^ It is, therefore, helpful to look at other indicators (e.g., overall access to health and medical facilities, percentage of cases receiving a microbiological diagnostic test, completeness of health facility reporting, antibiotic prescriptions) to interpret data. It may also be useful to examine other indicators, such as diagnostic test positivity rates.^10^

Despite these limitations, when used consistently across health services data, EMR can provide important surveillance data on disease burden, including populations at high risk, trends over time, geographic and seasonal patterns, and service utilization due to impetigo. The value of EMR data can be enhanced with the use of common case definitions, coding practices, and protocols for bacterial confirmation.

## Appendix 6: Quick List for Capturing Standardized Photographs

| **Step** | **Action** |
| --- | --- |
| 1 | Confirm all camera settings are correct* |
| 2 | Position participant in the shade: comfort, lesion exposed, neutral background |
| 3 | Photograph participant ID before capturing series or edit the photo if the device allows adding the participant ID |
| 4 | Position device in the same plane as sore, center the sore and focus camera; take additional photographs if none are clear and focused. It can be useful to have a closer picture to show the lesion, and another zoomed out to provide a sense of anatomical location |
| 5 | Record photograph number and notes in participant’s file |
| 6 | Save digital images in a secure location and delete them from the device |

*Recommended camera settings will depend on the device used and should be included as part of the surveillance program's SOPs.

Adapted from: Bowen et al. 2014 ^11^

## Appendix 7: Benefits from Working with Communities to Conduct Surveillance

| ***Purposeful surveillance:*** Development of a reciprocal approach that positively impacts the community. |
| --- |
| ***Build trust:*** Active inclusion and participation of communities can help build trust and facilitate effective and efficient implementation of surveillance activities. |
| ***Improve health literacy:*** Upskilling local health workers in recognizing and treating skin sores (impetigo) will improve the community’s health literacy and increase community capacity to manage these infections beyond the surveillance period. |
| ***Health promotion and awareness raising:*** In endemic settings specifically, changing community attitudes toward impetigo (i.e., changing the narrative) may help normalize skin sores. |
| ***Obtain valuable insight:*** Gain knowledge of local risk factors and contributors to impetigo and identify potential place-based strategies for impetigo prevention initiatives. |
| ***Improve accuracy of data collection and interpretation of results:*** Involving the community can assist in understanding language differences and meaning to improve the accuracy of data collection activities and contextualize the results. |
| ***Improve consent/participation rates:*** Involving community members in recruitment and the consent process can help facilitate trust and ensure adequate understanding for informed consent***.*** |
| ***Improve treatment/referral uptake:*** Using existing healthcare staff in surveillance can streamline the pathway to treatment and improve healthcare access and treatment uptake. |
| ***Future-proofing the project:*** Forming meaningful partnerships, upskilling and training community members interested in conducting surveillance, and involving communities in leading the surveillance will help maintain permissions for continued surveillance and community access. |

## Appendix 8: Terms to Describe Disease Burden

Incidence

***Incidence rates:*** An incidence rate is the number of new cases occurring per person per period of time at risk (person-time). The incidence rate is derived from the number of cases divided by the total number of person-time units (person-weeks, person-months, etc.) in which all the individuals were under surveillance. Person-time is the total time a person was observed as disease-free.

Note that data from children with lesions at the start of surveillance (i.e., prevalent cases) can only be included once they become disease-free, which can be aided by appropriate treatment. As Strep A impetigo is a common condition with a relatively short duration, incidence rates can be expressed as the number of cases per 100 child-years; however, enumerating cases per child-week enables examination of seasonal changes.

***Cumulative incidence:*** It is also possible to count incident cases over a specified period to determine the proportion of individuals in the population affected during that period. In this instance, the numerator is the number of people with a new episode during the surveillance period, and the denominator is the population at risk and free of impetigo at the beginning of the period. For example, if we survey 80 children in 1st grade for 12 months and detect a new episode of Strep A impetigo in 60 children, the cumulative incidence of Strep A impetigo in 1st graders is 75% for the 12 months. Often, children with recurrent episodes are only counted once.

Prevalence

There are advantages of using prevalence rather than incidence to measure disease burden from Strep A impetigo in endemic settings. Prevalence measures the number of people with impetigo at one specific instance in time (i.e., a particular day). All those with >1 impetigo lesion are counted in the numerator, and all at-risk people are counted in the denominator. This measure does not require investigators to define the beginning and end of an episode and offers economies of time and expense. However, because Strep A impetigo can have large seasonal variations, point prevalence measurements should be repeated periodically throughout the year.

## Appendix 9: Variables for Inclusion in Impetigo Surveillance Datasets

Clinical signs and symptoms of the disease in the past week: multiple choice fields are recommended. For impetigo, the following should be considered:

|  | **Required Variables** | **Optional Variables** |
| --- | --- | --- |
| **General** | - Participant unique ID number* - Date of enrolment | - Illness onset date |
| **Demographics** | - Age (in months if <1 year; otherwise in years) - Sex | - Date of birth - Race/ethnicity |
| **Clinical features** | - Presence of any purulent or crusted lesions | - Date of onset of lesions - Duration of symptoms (how long have you had them?) - Pain (do the skin sores hurt?) - Severity of symptoms - Current temperature - Concurrent sore throat |
|  | - Number of lesions (on upper and lower extremities and head/neck/scalp) | - Number of lesions on trunk/back - Location of lesions - Upper extremities - Lower extremities - Head/neck |
|  |  | - Type(s) of lesion(s) - Purulent - Crusted/scabbed - Flat/dry (healing) |
| **Epidemiologic features** |  | - Underlying skin disease (e.g., scabies, eczema, insect bites, trauma, herpetic outbreaks, other) - Exposure history to impetigo among family or household members, classmates, or other close contacts of the child - Number of household members - Number of bedrooms in household |
| **Treatment** |  | - Antibiotic (topical, oral, parenteral) prescribed at current health visit - Antibiotic dose - Antibiotic frequency - Duration of antibiotic use |
| **Microbiology** | - Participant unique ID number* - Specimen unique ID number - Specimen collection date - Date and hour plate is inoculated - Date and hour plate is placed in incubator - Date(s) and hour(s) plate is read - Name of reporting laboratory - Laboratory ID (if present)* - Group A *Streptococcus* identified: yes/no | - β-hemolytic Streptococcus identified: yes/no - If yes, choose one of the following groups: A, B, C, G, other - Other organism identified: yes/no - If yes, choose one: *Staphylococcus* *aureus*, other - Strep A identified from throat culture (serial, simultaneous collection of skin and throat specimens for culture may be performed to elucidate whether skin strains proceed to infect the throat or vice versa) - Date of notification of result to participant and/or doctor - Storage/transport identification number - Place/site of transfer of isolate for serologic or molecular typing and standardized antimicrobial drug susceptibility testing (i.e., reference laboratory) - Further testing ordered, e.g., emm typing, confirmation, further identification of large-colony β-hemolytic *Streptococcus* to the species or subspecies level, etc. |

*Enables linkage of laboratory data with participant, clinical and epidemiological data

ǂIf more than one specimen is taken, each specimen must have a unique ID number

## Appendix 10: Body Map to Record Skin Sores from Surveillance Assessments

**Document all skin infections on the body map below.**

For each skin lesion, please note the trigger when known. Mark the number of lesions in brackets at the end of coding and record the presence of any complications (e.g., 6.A.ii (3) N = there are three purulent sores on the left leg triggered by insect bites, with no complications observed).

If a swab has been taken, circle the number on the body part from where it was taken. Remember that if a child has multiple lesions, it is recommended that the most purulent lesion is swabbed.


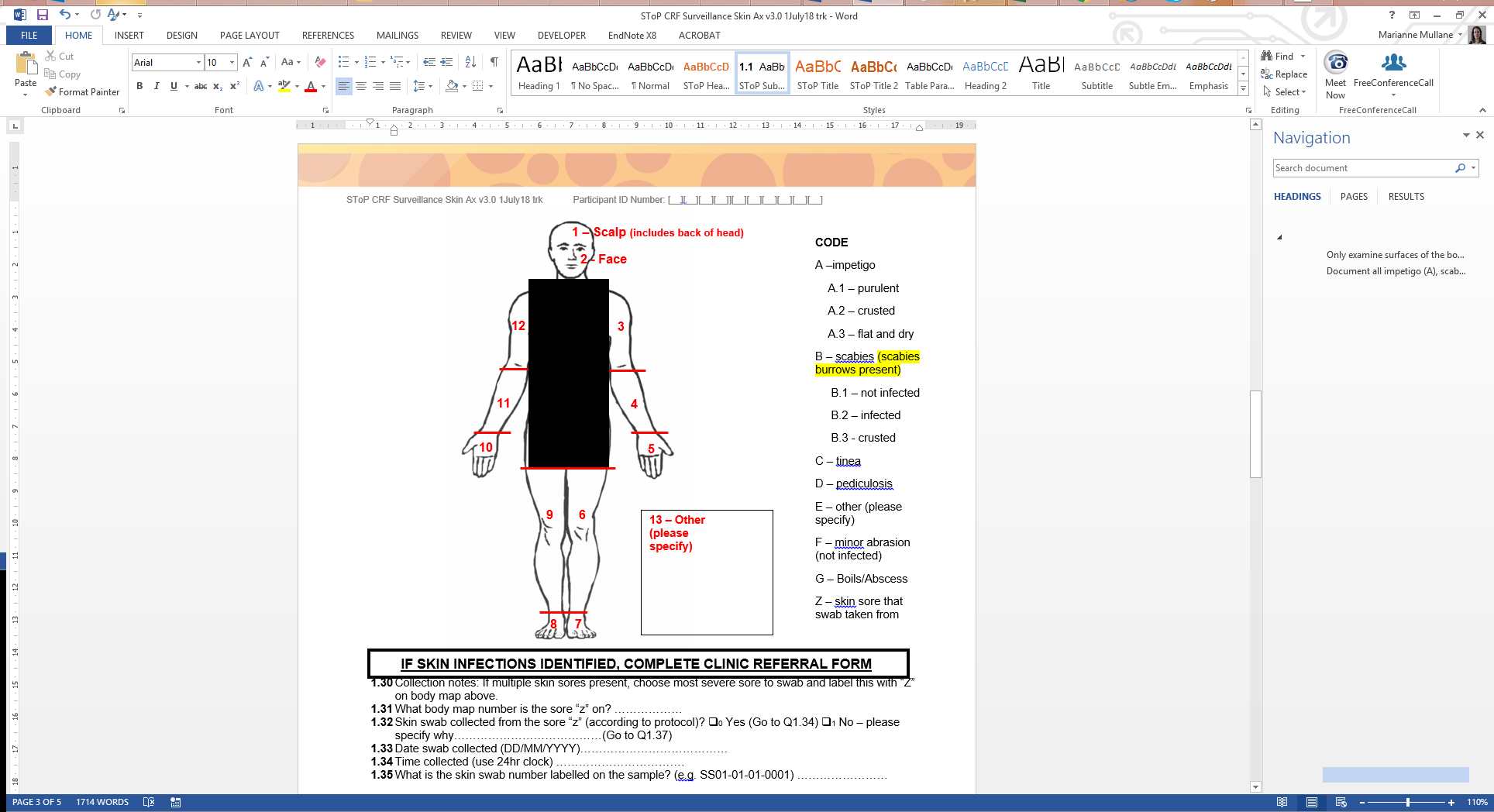


**CODING GUIDE**

**Body part**

1–12 (see body map)

Impetigo type

A. Purulent

B. Crusted

C. Flat and dry

**Trigger**

i. Minor trauma

ii. Insect bite

iii. Scabies

iv. Tinea

v. Head lice

vi. eczema

vii. Other (please specify)

**Complications**

N – No

Y – Yes (please specify i.e., abscess/boils, cellulitis)

(RIGHT)

(LEFT)

## References

1. World Health Organization. Surveillance standards for vaccine-preventable diseases. 2018.

2. World Health Organization. A definition for community-based surveillance and a way forward: results of the WHO global technical meeting, France, 26 to 28 June 2018. *Eurosurveillance.* 2019;24(2).

3. Deutscher M, Van Beneden C, Burton D, et al. Putting surveillance data into context: the role of health care utilization surveys in understanding population burden of pneumonia in developing countries. *Journal of Epidemiology and Global Health.* 2012;2(2):73-81.

4. U.S. Department of Health and Human Services. *E6(R2) Good Clinical Practice: Integrated Addendum to ICH E6(R1) Guidance for Industry* Maryland 2018.

5. Solares JRA, Raimondi FED, Zhu Y, et al. Deep learning for electronic health records: A comparative review of multiple deep neural architectures. *Journal of biomedical informatics.* 2020;101:103337.

6. Wang Z, Shah AD, Tate AR, Denaxas S, Shawe-Taylor J, Hemingway H. Extracting diagnoses and investigation results from unstructured text in electronic health records by semi-supervised machine learning. *PLoS One.* 2012;7(1):e30412.

7. Abdalla T, Hendrickx D, Fathima P, et al. Hospital admissions for skin infections among Western Australian children and adolescents from 1996 to 2012. *PloS One.* 2017;12(11):e0188803.

8. World Health Organization. *The International Statistical Classification of Diseases and Health Related Problems ICD-10: Tenth Revision. Volume 1: Tabular List.* Vol 1: World Health Organization; 2004.

9. Yeoh D, Anderson A, Cleland G, Banks A, Bowen A. Skin care Assessment in Broome and Port Hedland (SCAB HEAL) project. Paper presented at: International Congress for Tropical Medicine and Malaria. Brisbane, Australia 2016.

10. World Health Organization. Disease surveillance for malaria control: an operational manual. In. Geneva: World Health Organisation; 2012.

11. Bowen AC, Burns K, Tong SY, et al. Standardising and assessing digital images for use in clinical trials: a practical, reproducible method that blinds the assessor to treatment allocation. *PloS One.* 2014;9(11):e110395.
